# Chimpanzee (*Pan troglodytes*) gaze is conspicuous at ecologically-relevant distances

**DOI:** 10.1038/s41598-022-13273-3

**Published:** 2022-06-03

**Authors:** Will Whitham, Steven J. Schapiro, Jolyon Troscianko, Jessica L. Yorzinski

**Affiliations:** 1 Department of Ecology and Conservation Biology, Texas A&M University, College Station, TX USA; 2 Department of Comparative Medicine, UT MD Anderson Cancer Center, Bastrop, TX USA; 3 Centre for Ecology and Conservation, University of Exeter, Exeter, UK

**Keywords:** Behavioural ecology, Evolutionary ecology, Social evolution

## Abstract

Chimpanzee (*Pan troglodytes*) sclera appear much darker than the white sclera of human eyes, to such a degree that the direction of chimpanzee gaze may be concealed from conspecifics. Recent debate surrounding this topic has produced mixed results, with some evidence suggesting that (1) primate gaze is indeed concealed from their conspecifics, and (2) gaze colouration is among the suite of traits that distinguish uniquely social and cooperative humans from other primates (the cooperative eye hypothesis). Using a visual modelling approach that properly accounts for specific-specific vision, we reexamined this topic to estimate the extent to which chimpanzee eye coloration is discriminable. We photographed the faces of captive chimpanzees and quantified the discriminability of their pupil, iris, sclera, and surrounding skin. We considered biases of cameras, lighting conditions, and commercial photography software along with primate visual acuity, colour sensitivity, and discrimination ability. Our visual modeling of chimpanzee eye coloration suggests that chimpanzee gaze is visible to conspecifics at a range of distances (within approximately 10 m) appropriate for many species-typical behaviours. We also found that chimpanzee gaze is discriminable to the visual system of primates that chimpanzees prey upon, *Colobus* monkeys. Chimpanzee sclera colour does not effectively conceal gaze, and we discuss this result with regard to the cooperative eye hypothesis, the evolution of primate eye colouration, and methodological best practices for future primate visual ecology research.

## Introduction

The colours and patterns of primate faces are remarkably diverse, and are related to primate social ecology in ways that suggest adaptive, communicative functions^1^. For example, more varied, complex, and colourful facial patterning is most common in Old World primates that live in larger social groups^2^. Eye colours appear exemplary of this relationship between morphology and social ecology. The sizes, shapes, and colours of primate eyes are highly variable across species in ways that potentially affect their perceptibility and utility for communication^3^.

The cooperative eye hypothesis suggests that primate eyes are adapted to reveal or conceal gaze information^4^. According to this hypothesis, lighter primate sclera, like those of humans, are discriminable, conspicuous, and useful for gaze perception whereas darker primate sclera, like those of chimpanzees, are dark, obscured, and camouflaged in ways that impede gaze perception. The hypothesis suggests that these divergent phenotypes are the product of evolutionary pressures to cooperate and communicate prosocially (with highly discriminable gaze) or to avoid doing so (with eye colouration that conceals intraspecific cues), and that eye colouration thus provides a window into the evolutionary history of primate social ecology. In support of this hypothesis, natural human sclera colours are indeed highly perceptible, communicative cues. Newborn infants attend preferentially to individuals who gaze directly at them^5^, and exhibit rudimentary gaze following^6^. By 18-months-of-age, humans infants use gaze cues functionally to find hidden items^7^. Human adults detect gaze information more easily when sclera are naturally coloured, rather than darker or the same colour as the iris, across multiple light environments^8–10^. The extent to which dark sclera in nonhuman primates are cryptic is less clear. Chimpanzees have relatively dark sclera and follow the gaze of conspecifics in their social group^11^ as well as the gaze of a computerized conspecific on a computer monitor^12^. However, it is unclear in both of these cases whether chimpanzees use conspecific eyes specifically, rather than the head or body, to guide gaze following. More recent experimentation suggests that experimentally-enculturated chimpanzees are capable of discriminating computerized images of averted chimpanzee eyes from images of chimpanzee eyes directed forward, albeit with less success than when making the same discrimination of human eye images or chimpanzee eye images with reversed polarity^13^.

The cooperative eye hypothesis makes predictions about both primate eye colours and primate perception. One approach to studying the cooperative eye hypothesis measures primate eye colours, then estimates whether primates are likely to distinguish among those colours. For example, evidence that a species’ iris colors are highly discriminable from its sclera colors would suggest that the species is capable of using conspecific eyes as an informative cue in its social ecology. To this end, recent research has sought to quantify ape eye morphology using commercial photo-editing software and archival or publicly available photographs to estimate the relative conspicuousness of ape eye colours. Perea-García and colleagues reported that chimpanzee sclera are conspicuous, counter to the predictions of the cooperative eye hypothesis^14^. Mearing and Koops failed to replicate this result using the same method, reporting that chimpanzee and bonobo sclera are indeed cryptic^15^. Mearing and colleagues went on to report that phylogenetic analyses of 15 primate species suggested that patterns of scleral pigmentation (e.g., in chimpanzees) and depigmentation (e.g., in humans) were fundamentally related to species sociality, and were most likely to be functional adaptations for social cooperation or competition^16^. Other researchers have included the relative degree of scleral exposure, the width/height ratio of the eye, and other morphological measurements into such analyses. Mayhew & Gómez reported that the averted gaze of gorillas (*Gorilla gorilla*) reveals similar amounts of visible sclera as a human’s averted gaze, and is not heavily pigmented in many individuals^17^. Caspar and colleagues quantified ocular pigmentation and eye morphology across 15 hominoid and reported three unique phenotypic clusters: one that was uniquely human (bright, exposed sclera), one that included gibbons, siamangs, and chimpanzees (cryptic, hidden sclera), and one that included the remaining apes (variable pigmentation and morphology with brighter, more exposed sclera than the second cluster)^18^. Kano et al. performed sophisticated image analyses on images of seven great ape species and reported that whereas human eye outlines and iris colours were much more visually distinct in humans than other apes, ape eyes are broadly conspicuous rather than cryptic^19^.

Taken together, these studies demonstrate wide variability in eye morphology of primates, with uncertain functional significance. A key limitation of these studies (noted by^19^) is their omission of several key dimensions of animal perception. In particular, different species have different capacities to discriminate colour (e.g., sclera colours that are discriminable to human perception are not necessarily discriminable to other species) and to resolve fine detail (e.g., a given sclera may be conspicuous up to 5 m in one species but only up to 2 m in another species). And, all species’ ability to resolve fine detail decreases with increasing distance (e.g., sclera that are conspicuous at 5 m may be cryptic at 10 m within a given species)^20,21^. Furthermore, photographs of unknown origin and uncontrolled lighting conditions, and software that are tuned for human perception, are poor fits for empirical modeling of nonhuman animal perception^22,23^. Because any hypothesized relationship between ape eye morphology and social cognition depends on a conspecific observer’s ability to perceive and act on any cues the eye morphology produces, analyses that account for the unique perception of the observer species are a necessary part of understanding any hypothetical selection pressures for primate gaze colouration. For example, species-specific visual modeling reveals that the gaze colouration of a New World monkey species, *Sapajus apella*, is uniquely suited for signaling to conspecifics and prospective predators, but not to prey^24^.

**Figure 1:**
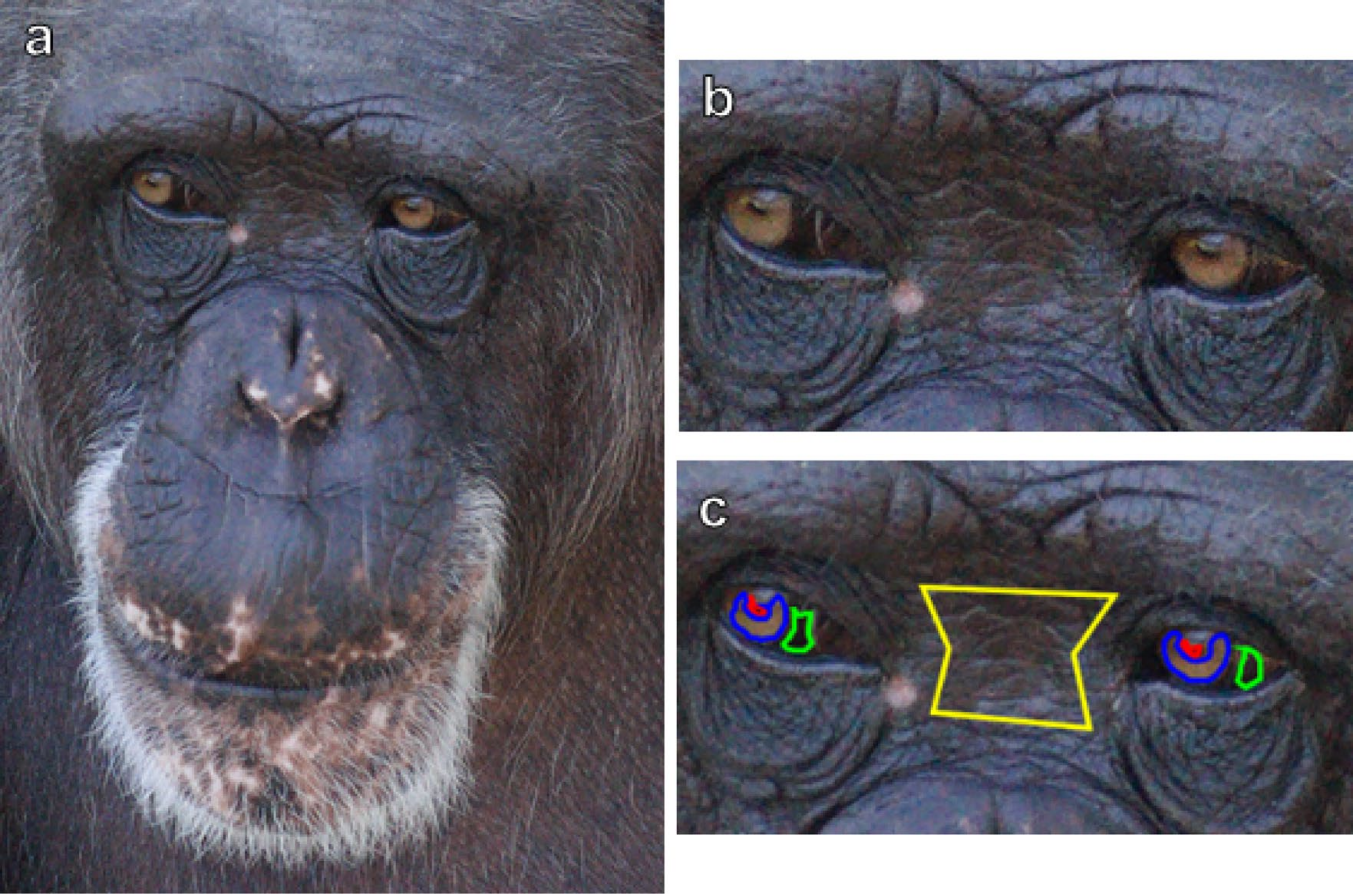
Chimpanzee face. (**a**) A sample photograph of a chimpanzee. (**b**) Enlarged view of the regions of the chimpanzee’s eye region. (**c**) Example ROIs for this photograph with pupil outlined in red, iris in blue, sclera in green, and skin in yellow. We omitted highlights in the ocular media, like the visible reflection of the sky, from all ROIs.

We tested the cooperative eye hypothesis using modern tools for modeling the species-specific and distance-dependent determinants of animal perception with colour calibrated photographs of chimpanzee faces. Specifically, we tested whether a perceptual model based on the chimpanzee visual system can discriminate among the colours of four regions of interest (ROIs) involved in perceiving gaze—chimpanzee iris, pupil, sclera, and skin (Fig. 1)—at simulated distances up to 16 m. Because the relative perceptibility of chimpanzee gaze may also affect chimpanzees’ facility in interspecific interactions, we also tested whether the visual phenotype of chimpanzees’ primate prey, the colobus monkey (genus *Colobus*), can perceive chimpanzee gaze information. In doing so, we offer a more complete account of how chimpanzee eye colour is perceived by chimpanzees and other primates.

## Results

**Figure 2:**
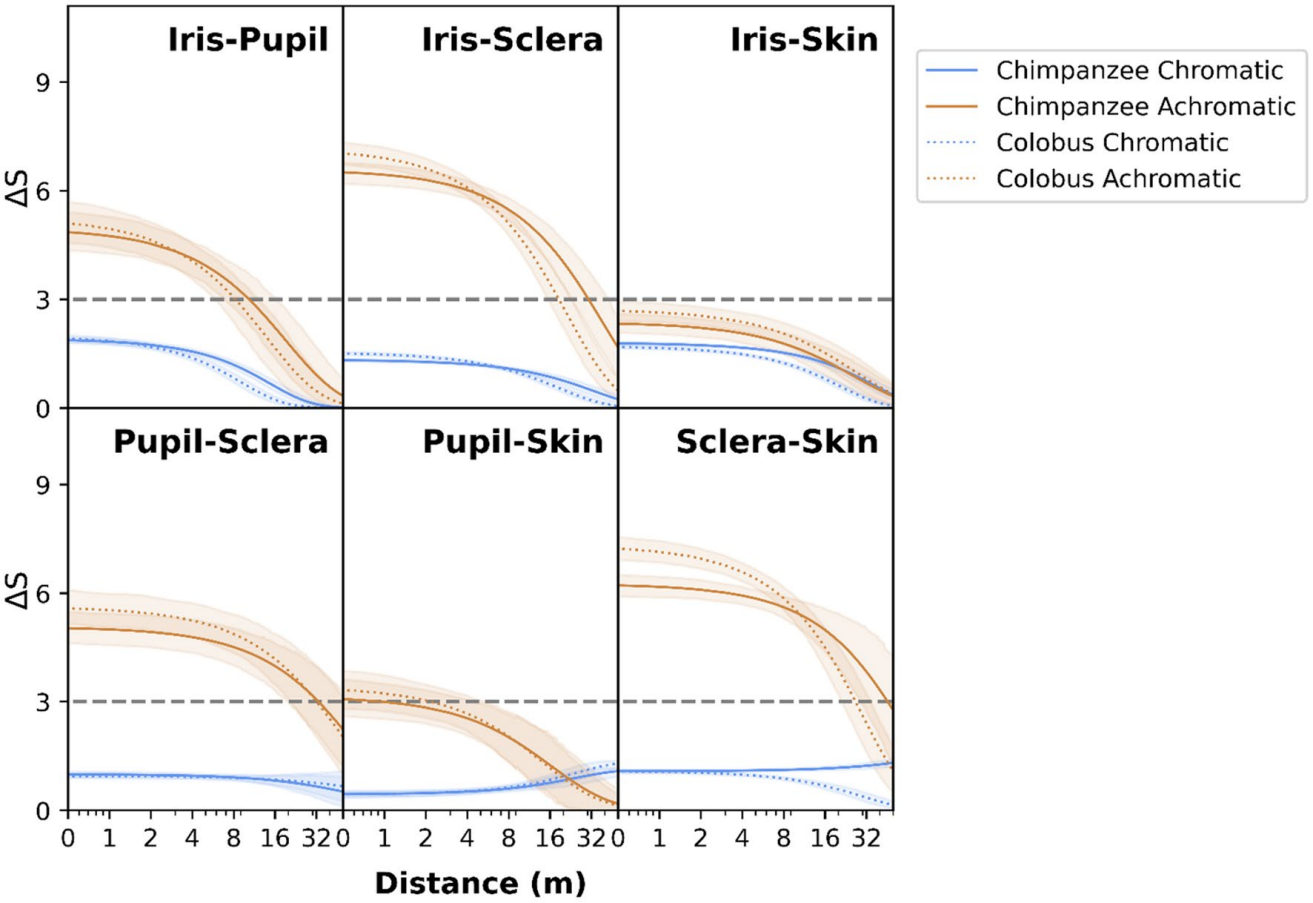
Chromatic and achromatic ∆S values for contrasts among the eye regions shaded in the top-right inset. A ∆S value greater than 3 at some distance suggest that the two ROI can be discriminated from each other. Shaded regions around lines are Bayesian 94% Highest Posterior Density intervals around ∆S estimates. Tick intervals on the x-axis are log-scaled.

Perceptual modeling suggests that the chromatic (color) information of chimpanzee iris, sclera, and pupil are not discriminable from each other at any distance (*∆S* discriminability measure values at all distances < threshold value 3), nor are they discriminable from chimpanzee face skin, for either species (Fig. 2). The achromatic (luminance) information of these same regions are discriminable from each other (*∆S* discriminability measure values > threshold value 3) by both species. Using the lower-bound of the highest posterior density interval as criteria for discrimination (see “Methods”), chimpanzees and colobus can discriminate the iris from the pupil using achromatic information when they are less than approximately 7 m and 6 m away, respectively. Similarly, chimpanzees can discriminate the sclera from the pupil, iris, or skin when they are approximately 20 m, 24 m, and 29 m away, respectively. And, colobus can discriminate the sclera from the pupil, iris, or hair when they are approximately 21 m, 16 m, and 21 m away, respectively. The ability of chimpanzees and colobus to discriminate ROIs is similar because they have similar color sensitivities and acuity (see “Methods”).

## Discussion

Using a visual modelling approach, regions of chimpanzee eyes relevant to gaze perception are discriminable from each other and from surrounding skin to both chimpanzees and colobus. Some discriminations (iris versus skin, and pupil versus skin) are difficult at any distance, while any discrimination involving chimpanzee sclera is likely possible at distances of 10 m or greater. While we do not know the specific facial regions that chimpanzees and colobus rely on to inform their gaze discriminations, our analyses suggest that they can use contrasts between the sclera and pupil, iris or surrounding skin to evaluate gaze. This result conforms to previous research in which human experimenters reliably identified chimpanzee gaze direction when they were within 10 m of the chimpanzees^25^. A 10–20 m distance at which chimpanzee sclera are conspicuous is likely to have many consequences for chimpanzee ecology. Many chimpanzee social behaviours—mating, fighting, grooming, feeding, play—necessarily occur within this distance. Outside this distance, it may be adaptive for gaze information to be more cryptic. Similarly, predators benefit by having their gaze cues hidden as they approach prey, as many prey species decide whether to remain in place or flee on the basis of predator gaze^26–28^. It would be adaptive for chimpanzee gaze cues to remain indiscriminable to *Colobus* monkeys as the apes approach, but the 10–20 m distance at which *Colobus* may discriminate chimpanzee gaze is likely beneficial to the monkeys*.* This dynamic perhaps underpins chimpanzee strategies for hunting *Colobus* that rely on coordinated pursuit rather than ambush^29^.

It is also probable that other determinants, aside from gaze perception, contribute to the evolution of facial colouration. The colours of primate hair and skin demonstrably vary with a primate’s native geography, climate, and diurnality; the relationship between these determinants and social ecology is inconsistent^1,2,30^. These non-social determinants of primate colour extend even to the regions immediately surrounding the eyes, with some Neotropical primates exhibiting dark eye masks that likely function as defense from UV radiation^1^. A phylogenetically-controlled analysis of eye colour across the primate order that uses proper animal colouration techniques could provide evidence that primate eye colour is broadly adapted to its social ecology. The cooperative eye hypothesis would be supported if lighter sclera colours were visible to conspecifics or predators at greater distances than darker sclera. Absent such evidence, hypotheses that lighter sclera colours are of great functional significance in intraspecific signaling, or that the evolution of human sclera suggests a uniquely hyper-cooperative evolutionary trajectory relative to other primates, are unsupported by data from primate eye colours.

Primate eyes vary along several morphological continua, and this extensive variability accordingly yields high variability in the contexts in which primate eyes and gaze directions are discriminable. Our study modeled and emphasized the distance-dependent and species-specific nature of visual perception. Future research may extend these techniques with additional context—of how perception of chimpanzee eye colours changes in different lighting environments, or of how discriminable the amount of visible sclera available during species-typical social interactions is likely to be. The cooperative eye hypothesis regards chimpanzee sclera as exemplary of cryptic eye colouration. Our analyses, along with recent work on the topic^13,14,19^ confirm that this is not so to the chimpanzee visual system, and offer substantive context using established tools for studying animal sensory ecology. Additional studies will be necessary to experimentally determine whether chimpanzees can discriminate gaze and at what distance they can make these discriminations, while accounting for their specific visual system.

## Methods

### Photograph collection

We photographed captive chimpanzees housed at The University of Texas’ MD Anderson Cancer Center's Michale E. Keeling Center for Comparative Medicine and Research in April and May of 2021. All work was approved by the Institutional Animal Care and Use Committee at the Keeling Center (IACUC approval number 0894-RN01), followed the guidelines of the Institute of Medicine on the use of chimpanzees in research, complied with the Society for Neuroscience Policy on Ethics, and reported in accordance with ARRIVE guidelines^31^. We used a Sony α7 II (ILCEm2) mirrorless digital camera (24 megapixel sensor) and Sony lens with 70–350 mm focal length for all photography. To standardize lighting as much as possible across photographs we photographed animals outside at a distance of approximately 10 m, in artificial shade, only on clear, sunny days (between 10:27 and 15:16) using the same aperture (f/6.3) and focal length (350 mm) for all photography. Because it was not feasible to place a photography reflectance standard in the same lighting conditions as unrestrained chimpanzees, we photographed a grey concrete region of the animals' enclosure in the same lighting as the animal moments after taking each photograph of an animal (i.e., the sequential method^32,33^. To identify the grey value appropriate for each concrete region of the animals' enclosure, we took additional photographs of the concrete alongside a 20% grey reflectance standard (Spectralon). A total of 76 photos (11 chimpanzees, 1–23 photos per chimpanzee) were used for all analyses. Skin and iris ROIs were identifiable in every photograph, sclera ROIs were identifiable in 73 photographs, and pupil ROIs were identifiable in 23 photographs (due primarily to highlights in the ocular media masking pupil colouration).

### Cone catch conversion and acuity correction

**Figure 3:**
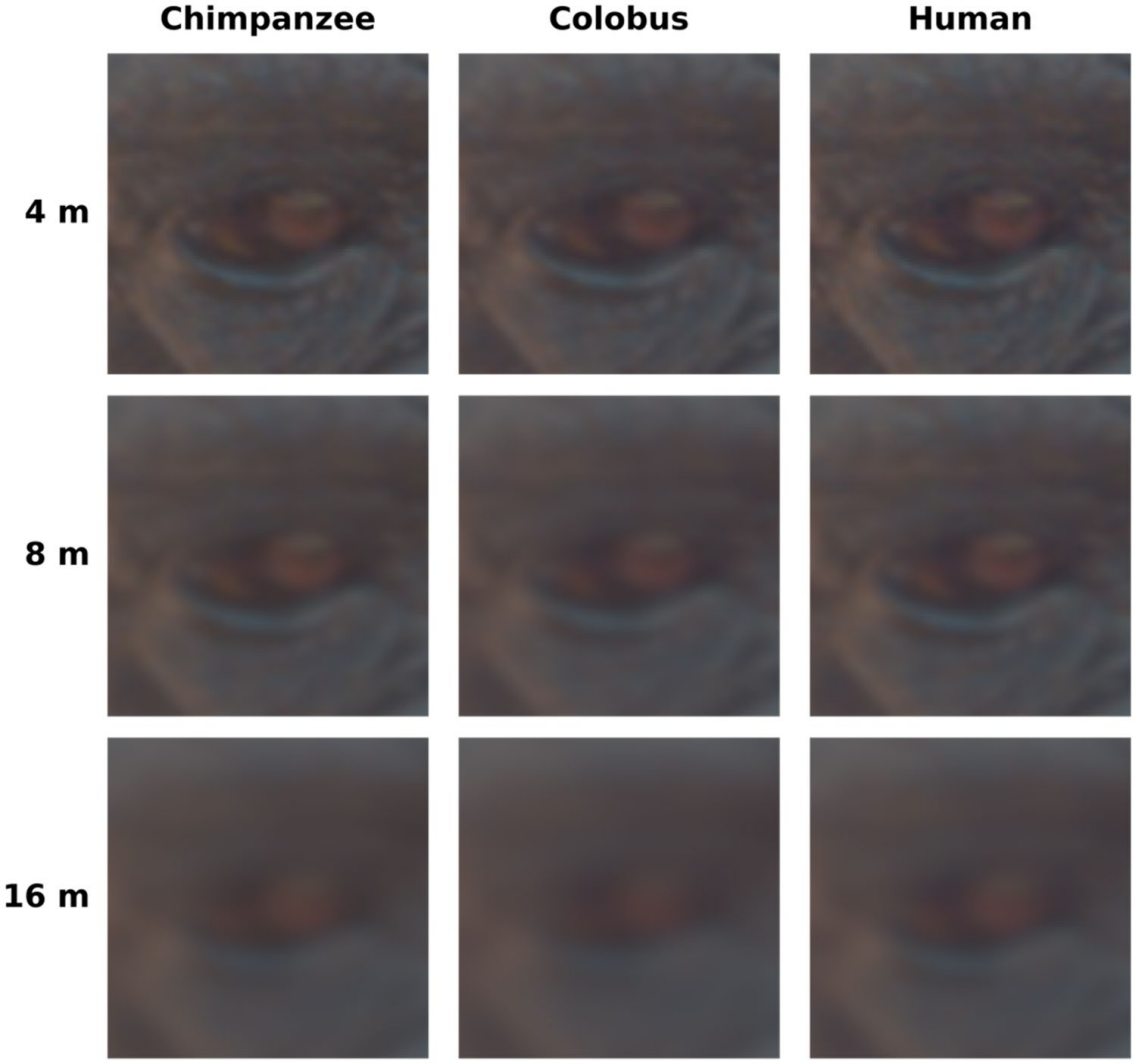
Visualizations of the effect of cone catch conversion and acuity correction steps on a chimpanzee eye as simulated in chimpanzee, colobus, and human vision phenotypes at simulated distances of 4, 8, and 16 m. The human vision phenotype is included only as a point of reference, and was not included in any analyses.

We used the micaToolbox (v.2.2.2) plugin for ImageJ (v.1.53e) to transform the colour and luminance data of the RAW photographs (.ARW format) into forms representative of chimpanzee and colobus colour vision^22^. Briefly, a linearized set of pixel values was extracted from each RAW photograph and normalized for differences in lighting using the concrete grey standard photograph taken immediately after each chimpanzee photograph. Then, the RGB sensitivities of our camera sensor (included in a stock version of micaToolbox) were mapped to chimpanzee and colobus monkey colour sensitivities using a linear regression, resulting in pixel values representative of the animals’ colour vision phenotype. We used the micaToolbox instantiation of AcuityView to transform photographs in ways representative of these species' vision at distances of 0.25, 0.5, 1, 2, 4, 8, and 16 m^20,34^; Fig. 3). The parameters of chimpanzee and colobus vision on which these operations are based, and relevant citations, are listed in Table 1.

**Table 1:** Parameters of chimpanzee and colobus vision.

	Wavelength of peak sensitivity for cone type				Receptor noise		
Phenotype	SW	MW	LW	Acuity (cyc/deg)	SW	MW	LW
Chimpanzee^a^	430	535	562	72	~ 0.229^†^	~ 0.057^††^	0.05
*Colobus*^b^	430	535	562	53.6	0.2	0.05	0.05

^†^0.22912878.

^††^0.0572822.

^a^Cone sensitivities from^35^; acuity estimate from^36^; receptor noise estimated with cone type ratio 1:16:21 for SW:MW:LW from^37^.

^b^Cone sensitivities from^38^ that suggested uniform colour vision among Old World monkeys; acuity from macaque estimate of^39^; receptor noise estimated with cone type ratio 1:16:16 for SW:MW:LW from^40^.

### Receptor noise limited modeling

We used chromatic^41^ and achromatic^42^ receptor noise limited (RNL) modeling procedures to estimate the discriminability of eye and face colours of the transformed chimpanzee photographs. This modeling yields psychometric distances (*∆S*) among regions of interest (ROI) that predict whether or not ROI are likely discriminable from each other. We used a conservative criterion of 3 *∆S* throughout, such that *∆S* > 3 are likely discriminable. Additional parameters of chimpanzee and colobus vision on which RNL depends are listed in Table 1.

### Statistical modeling

In order to quantify uncertainty around *∆S* values, we used *∆S* values to estimate 94% Highest Posterior Density intervals (HPD) using a Bayesian logistic regression with parameter estimates for each ROI contrast, vision phenotype, and distance. These HPD intervals estimate the likeliest 94% of values for an ROI contrast, and act as an easily interpreted decision criteria: if an HPD estimate for an ROI contrast is less than or includes 3 *∆S*, the ROI are plausibly not discriminable from each other. The regression also allowed for inferences about the effect of distance beyond the range of values that were explicitly modeled using AcuityView.

## Data Availability

All data that were used as regression model input are publicly available in an OSF repository at https://osf.io/ap74f/?view_only=3da59b82af3d4a9e9e4c200b958c53be.
